# Maximum Force Capacity of Back Extensor Muscles in Healthy Women and Men: Not Different, if Anthropometrically Normalized

**DOI:** 10.3390/jfmk11020212

**Published:** 2026-05-27

**Authors:** Christoph Anders, Beatrice Steiniger, Florian Saenger, Martin Marks, Lena Mader, Evgenij Dukvin, Anna Schneider

**Affiliations:** Division of Motor Research, Pathophysiology and Biomechanics, Experimental Trauma Surgery, Department for Hand, Reconstructive, and Trauma Surgery, Jena University Hospital, Friedrich-Schiller University Jena, Bachstraße 18, 07743 Jena, Germany; beatrice.steiniger@med.uni-jena.de (B.S.);

**Keywords:** healthy volunteers, female, male, maximum trunk extension force, upper body weight

## Abstract

**Background:** In this study, trunk extension strength was compared between healthy female and male participants. **Methods:** Overall, 115 women and 124 men underwent isometric maximal voluntary contraction (MVC) testing in an upright standing position. Upper body weight was additionally assessed. The primary outcome variables were maximal torque, upper body weight expressed as torque, and the MVC-to-upper-body-weight ratio. In view of the large sample size, interpretation of the results was based not only on statistical significance but also on clinical/practical relevance, as reflected by effect size (ES) and the relationship between the difference in mean values (DMV) and the minimal important difference (MID). Results were classified as relevant only when all three predefined criteria were met (*p* < 0.05, ES > 0.5, and DMV > MID). **Results:** Men showed significantly higher MVC values than women (241 Nm vs. 162 Nm; *p* < 0.0001; ES = 1.974; DMV > MID). Likewise, upper body torque was significantly greater in men than in women (115 Nm vs. 80 Nm; *p* < 0.0001; ES = 2.119; DMV > MID). However, after normalization for upper body torque, the between-sex difference in relative strength was no longer considered relevant (2.16 vs. 2.00; *p* = 0.0055; ES = 0.364; DMV < MID). **Conclusions:** These findings indicate that although absolute trunk extension strength differs markedly between sexes, relative strength adjusted for upper body weight is broadly comparable. Both women and men demonstrated a physiological strength reserve of approximately 100% relative to upper body weight.

## 1. Introduction

For many years, diagnostic data, therapeutic recommendations, and reference values, including those for laboratory parameters, were applied uniformly to all patients irrespective of sex. The recognition that such generalizations are inappropriate initially emerged in the context of laboratory medicine, where sex-specific reference ranges, particularly for hematological parameters, have been established for approximately 100 years. In contrast, other sex-specific laboratory parameters have only been introduced into routine practice over the past 30–40 years [1,2]. In clinical diagnostics, sex-specific symptom presentation is now also taken into account; a prominent example is the differing manifestation of myocardial infarction symptoms in men and women [2].

At present, sex-specific approaches to diagnosis and therapy have become the rule rather than the exception in medicine [3].

Sex-specific differences are particularly relevant for parameters associated with anthropometric characteristics [4] and physical performance [5]. Notably, however, the available literature does not indicate systematic sex differences in the fiber type composition of skeletal muscle [6,7]. Rather, individual factors, particularly genetic [8] and psychosocial factors, appear to play a decisive role.

Within this broadly comparable distribution of muscle fiber types, however, sex-related differences are evident. Men generally exhibit a larger muscle fiber cross-sectional area than women [7]. This difference is particularly pronounced in type II fibers: in men, type II fiber diameters are significantly greater than those of type I fibers, whereas such a distinction is not observed in women [6].

A similar pattern is observed in the anthropometric characteristics of healthy individuals: women are, on average, shorter and lighter than men [4,9]. Nevertheless, or perhaps precisely for this reason, no systematic sex differences in body mass index (BMI) have been identified [10]. However, sex-specific reference values are increasingly being recommended for this parameter as well, given that body composition differs between women and men [11] and is additionally influenced by age [11].

The function of the back, in conjunction with the abdominal muscles, is to provide an appropriate balance between spinal stability and mobility in order to prevent injury. In this context, three functional domains can be distinguished: maximal strength capacity, endurance capacity, and adequate coordination with regard to the timing of muscular activation [12,13], together with the aspect of intermuscular coordination.

Particularly with respect to maximal strength capacity, women appear to be at a disadvantage, as their values are estimated to be approximately 30% lower than those of men [14]. Adequate function of the entire trunk musculature is essential to maintain the necessary balance between spinal stability and mobility [12]. In particular, coordinative deficits of the abdominal muscles [15], as well as both coordinative impairments [16,17] and, especially, strength deficits [18] of the back muscles, have been associated with nonspecific low back pain. Accordingly, functional assessment of the trunk musculature represents a fundamental component of both the diagnostic evaluation and the effective treatment of low back pain.

Specifically, with regard to the aforementioned coordinative deficits of the abdominal muscles, it has been demonstrated that activation of these muscles normally occurs even prior to the initiation of planned movements, whereas such anticipatory activation could not be observed in patients with low back pain [19]. This preparatory activation is, however, necessary to generate adequate intra-abdominal pressure [20,21], which acts as a counterforce to the back muscles and thereby contributes to segmental stability [22,23,24]. Delayed activation in response to unexpected loads acting on the upper body has likewise been demonstrated [25].

Comparable findings of coordinative deficits have also been reported for the back muscles [26], along with impairments in fatigability [27] and force-generating capacity [18]. In this context, the function of the deep, segmentally stabilizing portions of the back muscles appears to be of particular importance [28]. These alterations are accompanied by changes in muscle fiber composition [29].

For many parameters characterized by substantial interindividual variability, normalization procedures are applied to reduce this variability [30]. Examples include the normalization of load-related measures to maximal strength [31] and to submaximal force values [32,33]. In addition to the previously discussed need for sex-specific analyses [14,34], force values are commonly normalized to anthropometric measures, particularly those related to body size [35]. In the present analysis, the measured force values had already been adjusted for differences in upper body length by conversion into torque values. A further question of interest, therefore, was whether additional information could be obtained by normalizing these values to upper body weight.

Accordingly, the aim of the present study was to investigate the extent to which such differences remain observable when strength values are normalized for anthropometric characteristics [36]. The primary outcome parameter was maximal trunk extensor force capacity normalized to upper body weight.

## 2. Methods

The present study comprises data from five previous investigations in which the same parameters were assessed. All studies were conducted in healthy voluntary participants of both sexes and were approved by the responsible ethics committee (4348-02/15, 2021-2373-BO, 2021-2373_1-BO, 2024-3462-BO, 2024-3634-BO-A). Written informed consent was obtained from all participants prior to participation. No participant was included in more than one study.

Overall, data from 115 women and 124 men aged 18–52 years were included in the present analysis. All participants were healthy, reported no acute back pain and no back pain within the preceding three months [37], and were either physically inactive or only moderately active [38]. Individuals engaging in regular physical exercise more than twice per week were excluded, as were those with a BMI > 32 kg/m^2^. The full anthropometric characteristics of the cohort are presented in Table 1.

### 2.1. Investigation

All measurements were performed using a computer-assisted testing and training device (CTT Centaur, BfMC, Leipzig, Germany). Within the device, participants stood in an upright position with the lower body fixed, while the upper body remained mobile. The system is equipped with a shoulder harness that is lowered onto the participants’ shoulders. Integrated force sensors within this harness allow for the measurement of forces in both the sagittal and frontal planes.

Maximal trunk extension force was assessed following a standardized warm-up protocol using submaximal loads. The MVC measurement itself was performed with a built-in program of the CTT Centaur and consisted of three consecutive trials, each of 5 s duration, separated by rest intervals of 5 s. Participants were instructed to reach maximal force within approximately 1 s, maintain this level for 2–3 s, and then relax. Standardized verbal encouragement was provided during each trial [39,40,41].

**Figure 1:**
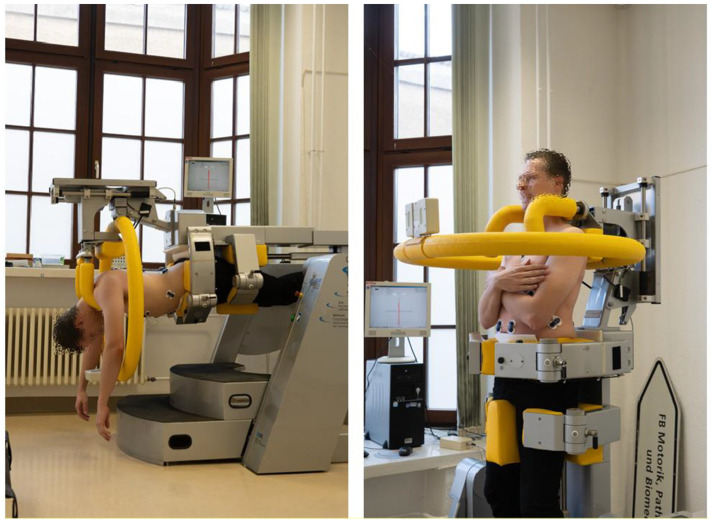
Investigation situation. **Left**: determination of upper body weight, **right**: subject performing isometric maximum extension force. Note that during all situations, participants remained in upright body position.

To familiarize participants with the procedure, the first trial was performed as a practice run at approximately 50% of maximal effort. During all measurements, participants crossed their arms in front of their chest (Figure 1).

In addition, upper body weight was assessed by tilting the CTT Centaur forward by 90° and positioning participants in a relaxed posture within the shoulder harness, with the arms and head hanging freely. In this position, upper body weight was recorded. The procedure was repeated three times, and the highest plausible value was used as the valid upper body weight (UBW) for further analysis. According to this procedure, the UBW measured in the present study comprised the torso, arms, and head [42].

The measured force values were converted into torque values, here referred to as upper body torque (UBT), in order to account for differences in body size and to provide directly comparable parameters for analysis.

### 2.2. Analyzed Parameters

The highest force value obtained across the three extension trials was defined as the maximum voluntary contraction (MVC). This parameter was analyzed both as force and, after conversion, as torque, termed maximum voluntary torque (MVT). Torque values were calculated using the distance between the horizontal projection of the scapular spine onto the spinal column and the horizontal level of the iliac crest as the lever arm length.

The measured maximal torque values were subsequently related to upper body weight, yielding the upper body torque ratio (UBTR).

### 2.3. Statistics

All data were tested for normal distribution using the Kolmogorov–Smirnov and Shapiro–Wilk tests. Normal distribution was confirmed for all variables.

Sex differences were analyzed using the independent-samples *t*-test. In addition, effect sizes (ES) were calculated for all comparisons [43].

To reduce the risk of Type I errors associated with the large sample size, the minimal important difference (MID) was additionally calculated as a mathematically defined measure (SD/2), as recommended in the literature, in order to identify differences considered relevant in relation to the difference in mean values (DMV) [44]. On this basis, an observed difference between compared values should be considered relevant only if the mean difference exceeds the MID [45]. Furthermore, with regard to effect size, differences were considered relevant only if ES was at least 0.5 [46].

Accordingly, only results meeting all three criteria (*p* < 0.05, ES > 0.5, and DMV > MID) were interpreted as relevant.

Independently of these group comparisons, the influence of sex on the analyzed parameters was examined using linear regression with “sex” entered as the predictor variable.

## 3. Results

**Figure 2:**
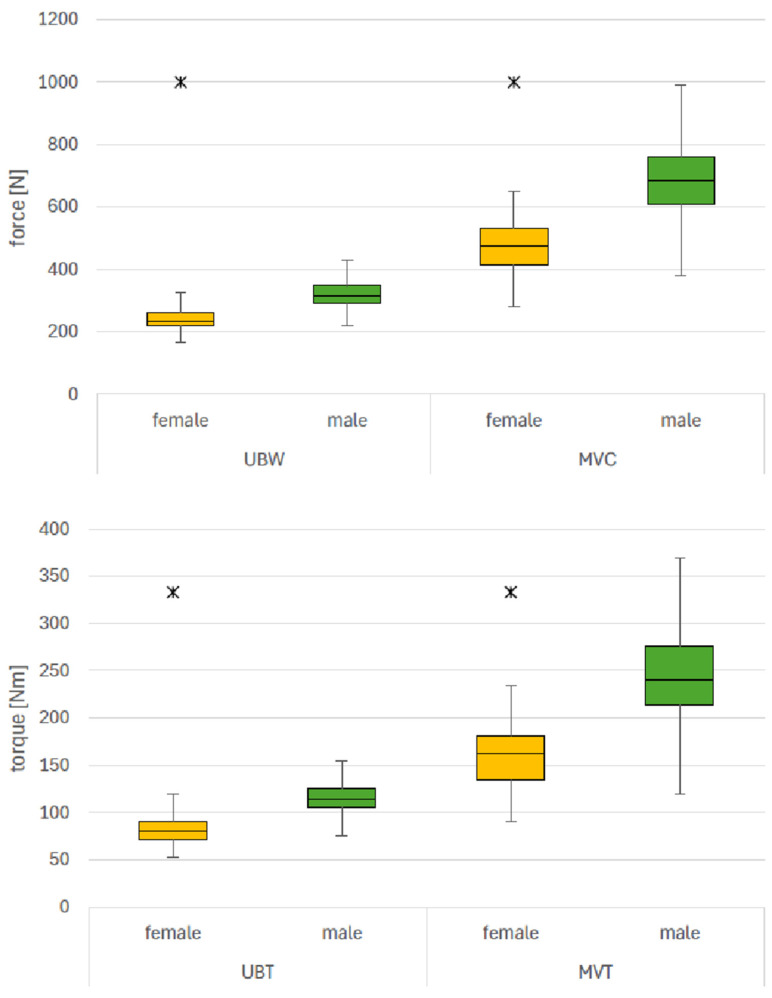
Measured data for upper body weight and maximum extension force. **Upper panel**: data provided as force values; **lower panel**: data provided as torque values. Asterisks indicate significant differences between sexes. UBW: upper body weight; MVC: maximum voluntary contraction force; UBT: upper body torque; MVT: maximum voluntary torque.

**Figure 3:**
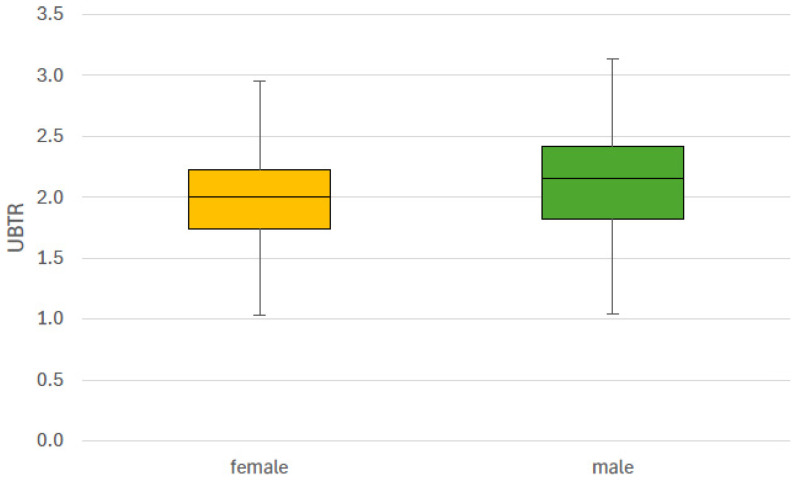
Upper body torque ratio (UBTR) for female and male subjects.

In the sex-based comparison, men showed significantly and relevantly higher values for UBW and MVC and for UBT and MVT (Figure 2). The mean UBTR values, together with their 95% confidence intervals, were 1.98 ± 0.07 for women and 2.12 ± 0.07 for men. Although a statistically significant sex difference was also observed for the UBTR (*p* = 0.0055), this finding was not considered relevant, as both ES < 0.5 and DMV < MID (Figure 3).

Detailed results of the statistical analyses are presented in Table 2.

Using sex as the predictor variable, the regression analysis showed that sex explained 54.3% of the variance in UBT, 49.5% of the variance in MVT, and only 2.8% of the variance in the UBTR.

## 4. Discussion

With regard to MVC and MVT, the results are not unexpected, as men are generally assumed to exhibit substantially greater maximal force than women [47]. This was reflected in the present data, in which maximal force values were approximately 45% higher in men than in women. Comparable findings have been consistently reported in the literature [14].

In contrast, upper body weight is only rarely measured in study populations. To date, there is virtually no standardized method that is simultaneously cost-effective, practical, and reliable [48,49,50]. By implementing specific modifications to the device used in the present study, it became possible to record force measurements in the tilting direction. In combination with the fixation of the lower body, this allowed upper body weight to be determined for all participants. In general, the device employed can be expected to yield reliable measurements [51].

Using this approach, individual upper body weight could be determined directly rather than estimated by modeling. Notably, relevant sex differences were also observed for this parameter, with men showing approximately 35% higher upper body weight than women. Since no normative data could be identified in the literature, these measurements already represent novel findings.

The results for the UBTR were somewhat unexpected: although the *p*-value indicated statistical significance, the difference was ultimately not considered relevant when effect size (0.364, i.e., small [43]) and the MID were taken into account, as the MID exceeded the mean difference.

Nevertheless, it should be noted that the confidence intervals did not include the respective mean values. However, confidence interval calculations are based on the standard error, which is strongly dependent on sample size [52,53]. Consequently, this estimate is subject to the same sample size-related influences as the test statistic itself.

Thus, both sexes appear to exhibit a comparable ratio of maximal trunk extension force to upper body weight. The existing differences in maximal force are offset by the considerably lower upper body weight in women. Consequently, the physiological reserve, that is, the remaining surplus of strength capacity, appears to be comparable in both sexes. Similar findings have been reported in the literature, where normalization to body weight likewise eliminated sex differences in MVC [54].

The results of the regression analysis clearly underline the strong dependence of UBT and MVT on sex while at the same time indicating that the UBTR is largely independent of sex.

The present data also provide preliminary reference values for the UBTR, amounting to approximately 2.0 in women and 2.1 in men. This suggests that, in both sexes, a physiological strength reserve of about 100% of upper body weight may be assumed. In addition, the calculated MID of approximately 0.2 may help us to identify meaningful deviations from these reference values and thereby support evidence-based recommendations for intervention when values fall below the expected range of UBTR variability [55].

Accordingly, future use of back muscle strength measurements may be considered diagnostically informative, with a performance capacity below 1.8 times upper body weight potentially indicating relevant deconditioning of the back muscles [56,57]. Since deconditioning of the back musculature is known to be associated with the occurrence of low back pain [18], the present findings may have diagnostic as well as therapeutic implications. These assumptions should, however, be examined in appropriately designed training intervention studies.

Markedly elevated UBTR values may also be of diagnostic interest, most likely when interpreted in relation to the force capacity of the abdominal muscles. In healthy adults, trunk flexor strength is reported to amount to approximately 0.6 to 0.8 of trunk extensor strength [58]. The extent to which not only absolute back muscle strength but also a balanced strength ratio between abdominal and back muscles is decisive for optimal trunk function in terms of stability and mobility should be addressed in future studies.

### Limitations

The investigations were carried out in a specialized testing environment. The device used (CTT Centaur) is not part of standard diagnostic or therapeutic equipment. Accordingly, the results should be interpreted with caution, as they may be device-specific. At the same time, all 239 participants were assessed using the same device, which strengthens the internal consistency of the observed sex differences.

For the determination of upper body weight, several model-based estimation approaches have been described [59,60]. However, in individual cases, direct measurement using appropriately positioned scales remains the preferable method [61].

Furthermore, no assessment of body composition was performed. Identical BMI values may be associated with substantially different body compositions [62,63]. To reduce this potential source of variability, individuals with a BMI > 32 kg/m^2^ and those engaging in physical exercise more than twice per week were excluded [64]. Nevertheless, future studies in groups with more heterogeneous levels of physical activity are warranted and should ideally include an assessment of body composition [65].

Finally, the statistical approach used to evaluate the findings may itself be open to discussion. Further studies are therefore needed to verify the conclusions drawn.

## 5. Conclusions

The present analysis showed that, despite sex-specific differences in the absolute strength of the back muscles, back muscle strength normalized to upper body weight does not differ meaningfully between men and women. In both sexes, the physiological strength reserve of the back muscles appears to correspond to approximately 100% of upper body weight.

## Figures and Tables

**Table 1:** Anthropometric characteristics of the participants.

	Height [cm]	Weight [kg]	Age [y]	BMI [kg/m^2^]
		Females (n = 115)		
MV	168.9	64.6	27.6	22.6
SD	6.4	9.6	8.0	3.1
CI	1.2	1.8	1.5	0.6
Median	169.0	63.0	24.0	22.1
upp. Q.	173.5	70.4	31.0	25.2
low. Q.	164.0	57.5	22.0	20.2
Males (n = 124)				
MV	180.8	78.5	30.7	24.0
SD	5.9	9.8	7.9	2.7
CI	1.0	1.7	1.4	0.5
Median	180.0	78.0	29.0	23.6
upp. Q.	185.0	84.6	37.0	25.8
low. Q.	177.0	72.5	24.0	22.2
Statistics				
*p*-value	<0.0001	<0.0001	0.0030	0.0003
ES	1.920	1.421	0.388	0.470
Relevance	relevant	relevant	Not relevant	Not relevant
Diff mean values	11.86	13.81	3.083	1.346
MID	3.09	4.86	3.977	1.432

MV: mean value. SD: standard deviation. CI: 95% confidence interval. upp. Q.: upper Quartile, low. Q.: lower Quartile; ES: effect size; MID: minimal important difference; Relevance: see Section 2.3.

**Table 2:** Detailed reporting of statistical results.

		UBT	MVT	UBTR
95% CI borders	Females	84.6/79.3 Nm	166.1/154.2 Nm	2.04/1.91
	Males	119.1/113.1 Nm	252.6/234.9 Nm	2.19/2.04
*p*-value		<0.0001	<0.0001	0.0055
ES		2.119	1.974	0.364
MID		8.19	21.48	0.195
Diff.MV		31.72	84.81	0.142
Rating		Relevant	Relevant	Not relevant

CI: Confidence interval; ES: effect size; MID: minimal important difference; UBT: upper body torque; MVT: maximum voluntary torque; UBTR: upper body torque ratio.

## Data Availability

The raw data supporting the conclusions of this article will be made available by the authors on request.
